# Delayed Nephropleural Fistula After Percutaneous Nephrolithotomy

**DOI:** 10.1089/cren.2016.0050

**Published:** 2016-05-01

**Authors:** Kamaljot S. Kaler, Daniel Cwikla, Ralph V. Clayman

**Affiliations:** Department of Urology, University of California, Irvine, Orange, California.

## Abstract

Pleural effusions due to pleural injury following supracostal percutaneous nephrolithotomy (PCNL) occur in upwards of 15% of patients; however, these effusions are invariably diagnosed immediately postoperative or during the hospital stay. Herein, we report our initial experience with a delayed nephropleural fistula. A 52-year-old female underwent an uneventful supracostal right PCNL staghorn stone procedure and was discharged on postoperative day 1. She presented to the emergency department 8 days after her original procedure and one day after ureteral stent removal in the office, with right pleural effusion, concomitant contralateral renal colic secondary to migration of a left pelvic stone into her left proximal ureter, and acute renal failure/oliguria. She was treated with right chest tube drainage, bilateral nephrostomy tube placement, and subsequent left holmium laser ureterolithotripsy.

## Introduction and Background

Percutaneous nephrolithotomy is the gold standard for the treatment of staghorn renal calculi. Obtaining optimal percutaneous access is essential in achieving a stone-free status. In general, upper pole access allows for a shorter tract and a direct path to the renal pelvis; however, this approach almost invariably requires a supracostal route with its attendant risk of an acute pleural effusion. These effusions are invariably diagnosed through intraoperative fluoroscopy or by a chest radiograph in the post anesthesia recovery room. The following case report describes an instance, in which the pleural effusion developed after the removal of the ureteral stent, 8 days postoperatively.

## Case Presentation

### Clinical history

A 52-year-old female presented to her primary care physician with hematuria, right flank pain, and suprapubic cramping. She underwent a CT scan of her abdomen and pelvis, which revealed a large right renal calculus and two smaller left renal calculi. She was subsequently referred to our office.

Her past medical history included hypertension, obesity, atrial fibrillation, rectocele, and dysfunctional uterine bleeding. Her surgical history included open cholecystectomy, two cesarean sections, and right-sided extracorporeal shockwave lithotripsy treatment in 1996. Neither stone analysis nor metabolic evaluation was available. Her family history is noncontributory in regard to nephrolithiasis. Her medications included metoprolol, aspirin, and sotalol. Her only allergy was to morphine.

Physical examination revealed a Caucasian female with a BMI of 47 kg/m^2^. She had a heart rate of 52, blood pressure of 160/75, and was afebrile. Abdominal examination revealed a soft, nondistended, nontender protuberant abdomen with a well-healed Pfannenstiel incision. The rest of her physical examination was unremarkable.

Urine analysis revealed a pH of 6, four white blood cells/high powered field, 73 red blood cells/high powered field, and no bacteria. Her urine culture showed growth of less than 10,000 CFU of alpha-hemolytic *Streptococcus* and diphtheroids. She was treated with ciprofloxacin for 7 days and tamsulosin for 2 days preoperatively before her procedure.

Laboratory studies revealed a white blood cell count 7400 cells/mcL, hemoglobin 13 g/dL, hematocrit 39%, and platelets 202,000 cells/mcL. Her sodium was 140 mmol/L, potassium 4.0 mmol/L, chloride 107 mmol/L, carbon dioxide 27 mmol/L, blood urea nitrate 16 mg/dL, and creatinine 0.7 mg/dL. Her calcium was 9.0 mg/dL and uric acid 5.0 mg/dL.

**FIG. 1.**
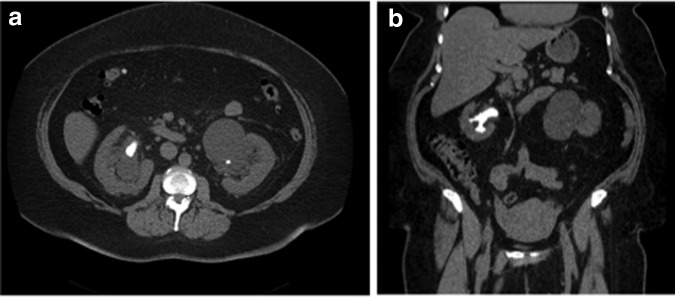
Preoperative axial and coronal stone protocol CT imaging. **(a)** Axial CT demonstrating presence of right staghorn calculus and left non-obstructing pelvic calculus. **(b)** Coronal CT demonstrating right staghorn calculus.

The initial CT scan revealed a right staghorn stone measuring 5.6 × 5.9 × 4.2 cm with low Hounsfield units (356–572). Her skin to stone distance ranged from 16 to 17 cm using axial images at 0°, 45°, and 90°. The stone filled the middle and lower calices, as well as the right renal pelvis, resulting in moderate hydronephrosis. Her left kidney contained two stones as follows: an 8 mm stone in the renal pelvis and a 2 mm upper pole stone. She also had a left peripelvic cyst measuring 7.8 × 8.1 × 8.3 cm, which was nonobstructing (Fig. 1).

### Surgical planning and treatment

**FIG. 2.**
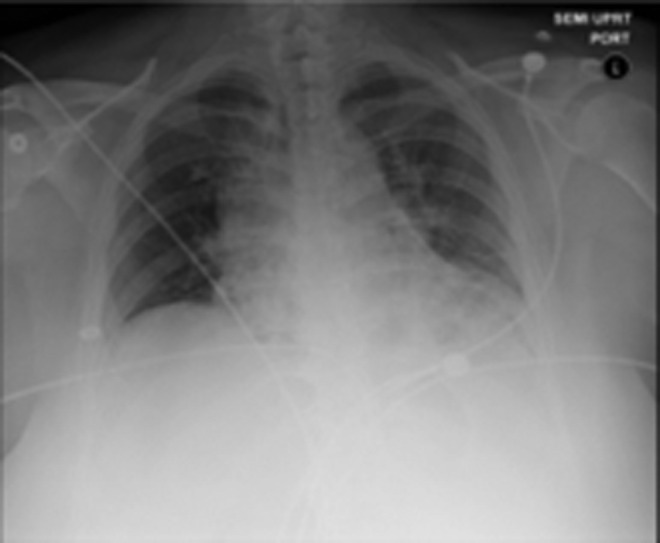
Postoperative chest X-ray performed immediately postoperatively demonstrated no evidence of intrathoracic fluid collection.

A right percutaneous nephrolithotomy through access of the right upper pole was planned; the proposed supracostal nephrostomy tract would be 16.3 cm (measured on sagittal films). With the patient prone on spreader bars, a 14/16F 55 cm ureteral access sheath was advanced retrograde over the initial guidewire. Under combined fluoroscopic and ureteroscopic control, a supra-11th rib access was obtained with a single pass of the nephrostomy needle. Using the holmium laser through the rigid nephroscope complemented by flexible ureteroscopy and flexible nephroscopy, the staghorn stone was cleared. At the end of the procedure, Surgiflo was used to seal the nephrostomy access using a 7F occlusion balloon catheter that was inflated at the junction of the collecting system and renal parenchyma. This positioning was confirmed before injection of Surgiflo using nephroscopy and fluoroscopy and left in place for 10 minutes. Finally, an indwelling 6F ureteral stent was placed. Overall, operative time was 4 hours with no intraoperative complications and estimated blood loss of less than 100 cc. A postoperative chest X-ray in the recovery room revealed no effusion and no pneumothorax (Fig. 2). She was discharged to home on postoperative day 1.

### Follow-up

The patient returned to our office 1 week later for stent removal. At that time, she was doing well and only complaining of mild right-sided discomfort felt to be related to her stent. Her laboratory investigations at that time revealed a creatinine of 1.4 mg/dL and blood urea nitrogen of 19 mg/dL. Serum electrolytes were normal. Her hemoglobin and white blood count remained unchanged compared with preoperative values. Stone analysis revealed 20% calcium oxalate monohydrate and 80% uric acid. Her stone culture demonstrated low counts of mixed flora, including mixed gram-negative rods, coagulase-negative Staphylococcus, and *Streptococcus viridans*. She underwent an uneventful stent removal in the office and was discharged to home.

One day after stent removal, she returned to the emergency department complaining of severe right flank pain, nausea, vomiting, and decreased urine output. In the interim, she also noted new onset left-sided pain. Laboratory investigations demonstrated an elevated creatinine of 2.6 mg/dL and a blood urea nitrogen of 25 mg/dL. Her blood glucose was 169 mg/dL. Compared with preoperative levels, her hemoglobin was slightly decreased at 10.3 g/dL with a hematocrit of 31% and platelets of 151,000 cells/mcL. A urinalysis revealed a pH of 6.0 with 153 white blood cells and 182 red blood cells/HPF and no bacteria. Her urine culture was negative.

**FIG. 3.**
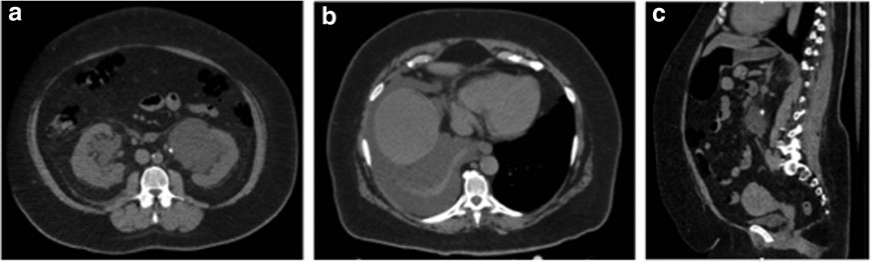
CT imaging performed in the emergency department. **(a)** Axial view demonstrating presence of left peripelvic cyst. **(b)** Axial view demonstrating large right-sided pleural effusion. **(c)** Sagittal view demonstrating obstructing left-sided ureteral calculus.

Upon admission, a CT scan was performed that showed a large right-sided pleural effusion and migration of her left renal pelvis stone into the proximal ureter. Due to her anatomy and the large left peripelvic cyst, the migration of the stone into the proximal ureter was only apparent on careful review of the sagittal views of the CT scan (Fig. 3).

With urine output of about 50 mL over 8 hours and her creatinine rising to 3.0 mg/dL, interventional radiology placed an 8F catheter to drain the right pleural effusion; 2 L of straw-colored fluid was drained immediately. Bilateral nephrostomy tubes were also placed by to relieve her obstruction and pain. The effusion had an elevated creatinine of 8.0 mg/dL. After the placement of the chest tube and the nephrostomy tubes, the patient showed marked improvement, with return of her creatinine to baseline over the next 3 days. Her chest tube was removed on the 3rd day and both nephrostomy tubes were left open to drainage. Interestingly, she continued to complain of intermittent left flank discomfort, although the left nephrostomy tube continued to drain well. She was discharged home on day 6. Two weeks later, the patient returned to the operating room for treatment of her left proximal stone. Interestingly, the coil of the left nephrostomy tube was limited within the peripelvic cyst with only a small opening communicating with the collecting system. This was incised at this procedure, and her left nephrostomy tube was maintained due to patient preference; no ureteral stent was placed on the left side. The right nephrostomy tube was removed after prompt excretion from her right collecting system.

The patient returned to our office 1 week later for routine postprocedural follow-up and a left nephrostogram. The nephrostogram showed prompt flow of contrast to the bladder; the left nephrostomy tube was removed. Her urine culture was negative. Subsequently, a full metabolic evaluation was completed, which showed low urine volume, hypercalciuria, and natriuria. She was placed on a low purine, low sodium diet, and advised to increase her fluid intake to 3–4 L/day.

## Discussion and Literature Review

Despite its prevalence and established place in treatment of large upper tract stone burdens, complications following percutaneous nephrolithotomy (PCNL) are not uncommon with a recent multicenter study showing a rate of ∼15 percent.^1^ Supracostal access to the renal collecting system has been reported to have higher morbidity due to a chance of pleural injury of 10%–30% for access above the 12th rib and 25%–35% for access above the 11th rib.^2–4^

Nephropleural fistula is a form of pulmonary injury following PCNL, in which a direct connection between the renal collecting system and thoracic cavity is created intraoperatively. Over time, drainage of fluid and urine into the chest occurs leading to pleural effusions and possible compromise of the patient's respiratory function.^5^ This communication is invariably recognized early either at the end of the procedure by performing fluoroscopy of the chest or in the immediate postoperative period on the chest radiograph obtained in the post anesthesia recovery room. The most definitive approach to this complication is immediate chest tube drainage.^2^

Although highly atypical, delayed presentation of nephropleural fistulas is a known entity, heralded by identical symptoms arising several days following the initial surgery. In one series, Munver and colleagues^5^ noted two cases of delayed nephropleural fistula from a total of 275 consecutive patients. Details regarding these cases, including the delay from the initial time of surgery and nature of presentation, are not provided, limiting comparison with our case.^2^ A second series of 375 consecutive patients by Lallas and coworkers^6^ presented a total of four nephropleural fistulas, two of which were detected immediately and two of which were delayed in presentation. The authors mention a single instance of nephropleural fistula where the patient was discharged home after an uneventful postoperative course, but returned 1 week later with evidence of a large pleural effusion. The authors suggest that this process may be related either to a slow leak from the kidney or an acute obstructive event (e.g., a blood clot or stone fragments) leading to tissue reopening of the nephrostomy tract and its communication with the pleural cavity.^6^ In both series, the authors state that all patients undergoing either supracostal or upper pole access underwent a chest X-ray postoperatively to rule out pneumothorax, hydrothorax, or pleural effusion.

Herein, we report an instance of a delayed nephropleural fistula developing despite a negative postoperative chest X-ray. Similar to previous occurrences, supracostal access to the kidney was obtained, with entry in this case occurring above the 11th rib. Although the patient initially did well with an uncomplicated hospital and postdischarge course, following ureteral stent removal on her 8th postoperative day, she returned within 24 hours complaining of respiratory symptoms. As her CT scan was negative for any right renal stone fragments, it is possible that she had ureteral edema, possibly transient following stent removal, blood clot, stone fragment, or even some Surgiflo that caused her obstruction.

## Conclusion

This case highlights an example when a delayed nephropleural fistula should be considered following an uneventful PCNL. In patients returning with flank discomfort and respiratory symptoms following stent removal, a delayed nephropleural fistula should be considered as part of the differential diagnosis.

## Abbreviations Used

CT: computed tomography
PCNL: percutaneous nephrolithotomy
